# Alcohol-Related Risk of Suicidal Ideation, Suicide Attempt, and Completed Suicide: A Meta-Analysis

**DOI:** 10.1371/journal.pone.0126870

**Published:** 2015-05-20

**Authors:** Nahid Darvishi, Mehran Farhadi, Tahereh Haghtalab, Jalal Poorolajal

**Affiliations:** 1 Department of Clinical Psychology, Hamadan Branch, Islamic Azad University, Hamadan, Iran; 2 Department of Psychology, Faculty of Economics and Social Sciences, Bu-Ali Sina University Hamadan, Iran; 3 Modeling of Noncommunicable Diseases Research Center, Department of Epidemiology & Biostatistics, School of Public Health, Hamadan University of Medical Sciences, Hamadan, Iran; University of Vienna, AUSTRIA

## Abstract

**Background:**

Several original studies have investigated the effect of alcohol use disorder (AUD) on suicidal thought and behavior, but there are serious discrepancies across the studies. Thus, a systematic assessment of the association between AUD and suicide is required.

**Methods:**

We searched PubMed, Web of Science, and Scopus until February 2015. We also searched the Psycinfo web site and journals and contacted authors. We included observational (cohort, case-control, and cross-sectional) studies addressing the association between AUD and suicide. The exposure of interest was AUD. The primary outcomes were suicidal ideation, suicide attempt, and completed suicide. We assessed heterogeneity using Q-test and I^2^ statistic. We explored publication bias using the Egger's and Begg's tests and funnel plot. We meta-analyzed the data with the random-effects models. For each outcome we calculated the overall odds ratio (OR) or risk ratio (RR) with 95% confidence intervals (CI).

**Results:**

We included 31 out of 8548 retrieved studies, with 420,732 participants. There was a significant association between AUD and suicidal ideation (OR=1.86; 95% CI: 1.38, 2.35), suicide attempt (OR=3.13; 95% CI: 2.45, 3.81); and completed suicide (OR=2.59; 95% CI: 1.95, 3.23 and RR=1.74; 95% CI: 1.26, 2.21). There was a significant heterogeneity among the studies, but little concern to the presence of publication bias.

**Conclusions:**

There is sufficient evidence that AUD significantly increases the risk of suicidal ideation, suicide attempt, and completed suicide. Therefore, AUD can be considered an important predictor of suicide and a great source of premature death.

## Introduction

Suicide is one of the top 20 leading causes of death in the world for all ages [1], the third leading cause of death among people aged 15–44 years, and the second leading cause of death among people aged 10–24 years [2]. These numbers underestimate the problem and do not include suicide attempts which are up to 20 times more frequent than completed suicide [2]. Furthermore, many people who have suicidal thoughts never seek services [3].

Suicide is among the greatest sources of premature death [4]. The number of people die from homicide and suicide is much more than the number of people die from the attack in a war. In fact, for every death due to war, there are three deaths due to homicide and five deaths due to suicide [5]. It is estimated that about one million people die annually from suicide, i.e., a global mortality rate of 16 per 100,000, or one death every 40 seconds [2].

There is no single cause of suicide. Suicide is complex with several psychological, social, biological, cultural, and environmental factors [2,6,7]. Alcohol and drug abuse are among the major risk factors for suicide [1,3]. The harmful use of alcohol is a global problem, which is associated with many serious individual and social consequences. In addition to the chronic diseases that may develop in those who drink large amounts of alcohol over a long period of time, a significant proportion of the disease burden is the result of intentional and unintentional injuries, such as violent behaviors, suicides, and traffic accidents [8].

Several reviews have discussed the relationship between alcohol use disorder (AUD) and suicidal thoughts and behavior, but none has given a pooled effect estimate [9–11]. An old meta-analysis was conducted by Smith et al [12] based on studies published before 1999. They measured blood alcohol concentration among unintentional injury deaths as well as homicide and suicide cases and concluded that blood alcohol concentration was high among the victims. However, no pooled estimate of the association between suicide and AUD was reported. Another meta-analysis conducted by Fazel et al in 2008 [13] to estimate the alcohol-related risk of completed suicide in prisoners. The results of this meta-analysis was limited to a specific population which may not be generalized to the general population. Furthermore, the association between AUD and suicidal ideation and suicide attempt was not investigated either.

Current evidence based on epidemiological studies has shown that AUD is associated with an increased risk of suicide, but there are serious discrepancies across the studies. Therefore, a systematic assessment of the association between AUD and suicide is needed. This meta-analysis was carried out to estimate the association between AUD and suicidal ideation, suicide attempt, and completed suicide separately.

## Materials and Methods

### Criteria for including studies

The supporting PRISMA checklist of this review is available as supporting information; see S1 PRISMA Checklist. Cohort, case-control, and cross-sectional studies addressing the association between AUD and suicide were included irrespective of participants' age, gender, language, nationality, race, religion, or publication status (published as a full text article in a journal or presented as an abstract at a conference). The observational studies addressing suicide rate among alcohol abusers without comparison group or self-harm without suicide intention were excluded.

The exposure of interest was AUD including alcohol abuse and alcohol dependence [14]. AUD is a condition characterized by the harmful consequences of recurrent alcohol use and physiological dependence on alcohol resulting in harm to physical and mental health and impairment of social and occupational activities [15]. The studies addressing the association between AUD and suicide among drug abusers or among patients with mental disorders were excluded.

The primary outcomes were suicidal ideation, suicide attempt, and completed suicide. A suicidal ideation is "thinking about, considering, or planning for suicide" [16]. A suicide attempt is "a non-fatal self-directed potentially injurious behavior with any intent to die as a result of the behavior" [16]. A completed suicide is "a death caused by self-directed injurious behavior with any intent to die as a result of the behavior" [16]. The death due to overdose without intent to die was not included. The studies reporting suicide as a general term without distinguishing between suicidal ideation, suicide attempt, or completed suicide were excluded.

### Search methods

The search strategy was as follows: (suicide or suicidal or suiciding or self-injurious behavior or self-immolation or self-destruction or self-slaughter or self-mutilation or self-harm or self-inflicted or self-injury) and (alcohol or alcoholic or alcoholism or harmful drinking or dependent drinking or over-drinking or harmful drinker or heavy drinker or hard drinker or problem drinker or pathological drinker or ethanol) and (cohort stud* or follow-up stud* or longitudinal stud* or case-control stud* or case-base stud* or cross-sectional stud* or observational stud* or survey).

The main bibliographic databases, including PubMed, Scopus, and Web of Science, were searched until February 2015. The reference lists of all included studies were scanned and the authors of the identified studies were contacted for additional eligible studies. The Psycinfo web site was searched as well.

### Data collection and analysis

Two authors (ND and JP) independently screened the title and abstract of the retrieved studies and decided on which studies met the inclusion criteria of this meta-analysis. The between authors disagreements were resolved through discussion among the authors until consensus was reached, otherwise a senior author arbitrated.

An electronic data sheet was developed and used for data extraction. Two authors (ND and JP) extracted data independently. The between authors disagreements were resolved through discussion among the authors until consensus was reached, otherwise a senior author arbitrated. The following data were collected: first author’s name, year of publication, country, mean age, gender, type of population (general or conscripts/veterans), study design (cohort, case-control, cross-sectional), suicide (ideation, attempt, completed), sample size, effect estimate with associated 95% confidence interval (CI).

The quality of reporting and the risk of bias of the included studies was explored using Newcastle Ottawa Statement Manual [17]. The scale allocates a maximum of nine stars for quality of selection, comparability, exposure and outcome of the study participants. The studies with seven star-items or more were considered a low risk of bias and those with six star-items or fewer were considered a high risk of bias.

Heterogeneity was explored using Q-test [18] and its quantity was measured using the I^2^ statistic [19]. Publication bias was assessed using the Egger's [20] and Begg's [21] tests and visualized by the funnel plot.

Measures of alcohol effect were expressed as risk ratio (RR) and odds ratio (OR). RR is the relative incidence risk of events in the exposed group versus the non-exposed group occurring at any given point in time. OR is the relative odds of outcome in the exposed group versus the non-exposed group occurring at any given point in time. Wherever reported, we used full adjusted forms of RR and OR controlled for at least one or more potential confounding factors such as age, gender, race, mental disorder, drug abuse, smoking, marital status, body mass index, educational level, employment status, income, and living alone.

Data were analyzed and the results were reported using a random effects model [22]. In order to explore the source of heterogeneity, we performed meta-regression analysis considering mean age, gender (percent of men), adjusted/unadjusted effect estimates, and a high/low risk of bias as covariates. All statistical analyses were performed at a significance level of 0.05 using Stata software, version 11 (StataCorp, College Station, TX, USA).

## Results

### Description of studies

**Fig 1:**
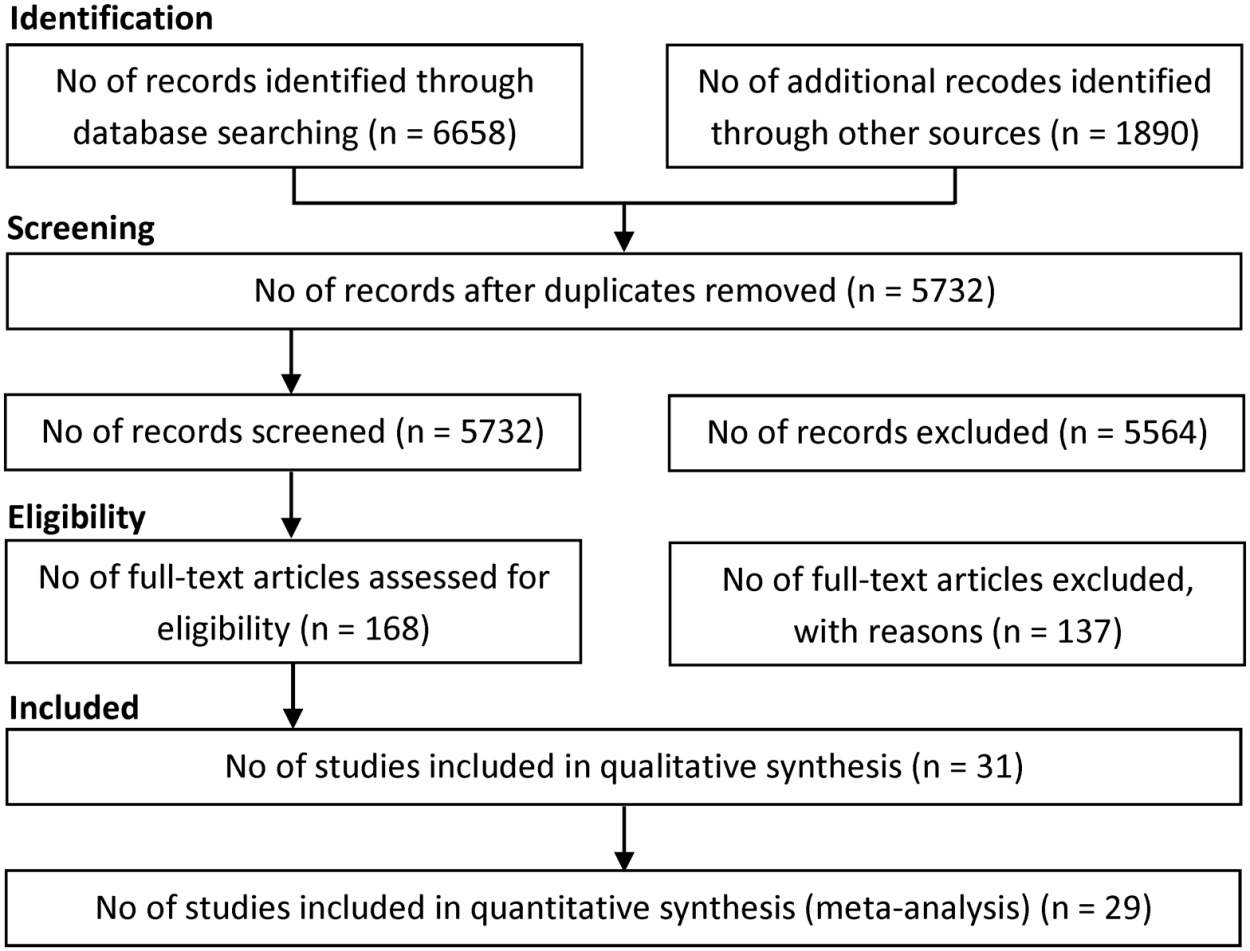
Flow of information through the different phases of the systematic review.

We retrieved 8548 references until February 2015, including 6658 references through searching electronic databases, 1890 references through checking other sources, including reference lists, relevant web sites, or personal contact with authors of the included studies. We excluded 8380 duplicates and clearly irrelevant references through reading titles and abstracts. Of the 168 references considered potentially eligible after screening, 137 studies were excluded because they were not original article (i.e., letter, commentary, review) or did not meet the inclusion criteria (Fig 1). Eventually, 31 studies included in the meta-analysis, including 9 cohort studies [23–31] and 10 case-control studies [32–41] and 12 cross-sectional studies [42–53]. All included studies were published in English. In cases of multiple publication, the last report was used.

The characteristics of the included studies are summarized and listed in Table 1. The included studies involved 420,732 participants. A study [52] assessed the association between AUD and suicide in two different countries (the USA and France) concurrently. Thus, this study is presented twice in Table 1 as well as the forest plots. Eight studies reported the association between AUD and suicidal ideation, 15 studies reported the association between AUD and suicide attempt, and 14 studies reported the association between AUD and completed suicide. Since four studies [29,30,46,52] have reported the association between AUD and suicidal ideation, suicide attempts, and completed suicide concurrently, therefore, the number effect sizes given in the forest plots is more than the total number of included studies. Some cohort studies reported RR and some others as well as the case-control and cross-sectional studies reported OR.

**Table 1:** Summary of studies results.

								Newcastle Ottawa Score		
1^st^ author	Country	Age	Gender	Population	Study	Estimate	Sample	Sel	Com	E/O
Agrawal 2013	USA	18–27	Female	General	Cross-sectional	Crude	3,787	****	*	**
Akechi 2006	Japan	40–69	Male	General	Cohort	Adjusted	43,383	****	**	***
Andreasson 1991	Sweden	18–21	Male	General	Cohort	Adjusted	49,464	***	**	**
Aseltine 2009	USA	11–19	Both	General	Cross-sectional	Adjusted	32,217	**	**	**
Bagge 2013	USA	18–64	Both	General	Case-Control	Adjusted	192	****	**	**
Beck 1989	USA	29.9	Both	General	Case-Control	Adjusted	413	***	**	**
Bernal 2007	Europe	18+	Both	General	Cross-sectional	Adjusted	21,425	***	**	**
Bunevicius 2014	Lithuania	18–89	Both	General	Cross-sectional	Adjusted	998	***	**	**
Coelho 2010	Brazil	18+	Both	General	Cross-sectional	Adjusted	1,464	**	**	**
Donald 2006	Australia	18–24	Both	General	Case-Control	Adjusted	380	****	**	**
Elizabeth 2009	USA	13–18	Both	General	Cross-sectional	Crude	31,953	**	*	*
Feodor 2014	Denmark	16+	Both	General	Cohort	Adjusted	32,010	****	**	***
Flensborg 2009	Denmark	20–93	Both	General	Cohort	Adjusted	18,146	****	**	***
Grossman 1991	USA	14.4	Both	General	Cross-sectional	Adjusted	6,637	***	**	***
Gururaj 2004	India	15–60	Both	General	Case-Control	Crude	538	****	*	**
Kaslow 2000	USA	18–64	Female	General	Case-Control	Crude	285	***	*	**
Kettl 1993	Alaska	30.8	Both	General	Case-Control	Crude	66	**	*	***
Lesage 1994	Canada	18–35	Male	General	Case-Control	Crude	150	****	*	***
Méan 2005	Switzerland	16–21	Both	General	Cohort	Adjusted	148	***	**	*
Morin 2013	Sweden	70–91	Both	General	Case-Control	Adjusted	515	***	**	**
Orui 2011	Japan	20+	Both	General	Cross-sectional	Adjusted	770	***	**	**
Petronis 1990	USA	Adults	Both	General	Cohort	Adjusted	13,673	FTU	FTU	FTU
Pridemore 2013	Russia	25–54	Male	General	Case-Control	Adjusted	1,640	****	**	**
Randall 2014	Benin	12–16	Both	General	Cross-sectional	Adjusted	2,690	**	**	**
Rossow 1995	Norway	19+	Male	Conscripts	Cohort	Crude	41,399	****	*	***
Rossow 1999	Sweden	Middle-aged	Male	General	Cohort	Adjusted	46,490	****	**	***
Shoval 2014	Israel	21–45	Both	General	Cross-sectional	Adjusted	1,237	***	**	**
Swahn 2012	France	19-Nov	Both	General	Cross-sectional	Adjusted	13,187	**	**	**
Swahn 2012	USA	19-Nov	Both	General	Cross-sectional	Adjusted	15,136	**	**	**
Tidemalm 2008	Sweden	37.7	Both	General	Cohort	Adjusted	39,685	****	**	***
Zhang 2010	China	34–60	Male	General	Cross-sectional	Adjusted	454	**	**	**
Zonda 2006	Hungary	52.1	Both	General	Case-Control	Crude	200	***	*	**

**Sel**: Selection; **Com**: Comparability; **E/O**: Exposure/Outcome; **FTU**: Full text unavailable

**Adjusted** means controlled for one or more of the following factors: age, gender, race, mental disorder, drug abuse, smoking, marital status, body mass index, educational level, employment status, income, living alone

The results of assessing risk of bias of the included studies are given in Table 1 based on the Newcastle Ottawa Statement Manual. Based on this manual, 10 studies had a high risk of bias and 20 studies had a low risk of bias. A study [28] had no full text and thus its risk of bias was not evaluated.

### Effect of exposure

**Fig 2:**
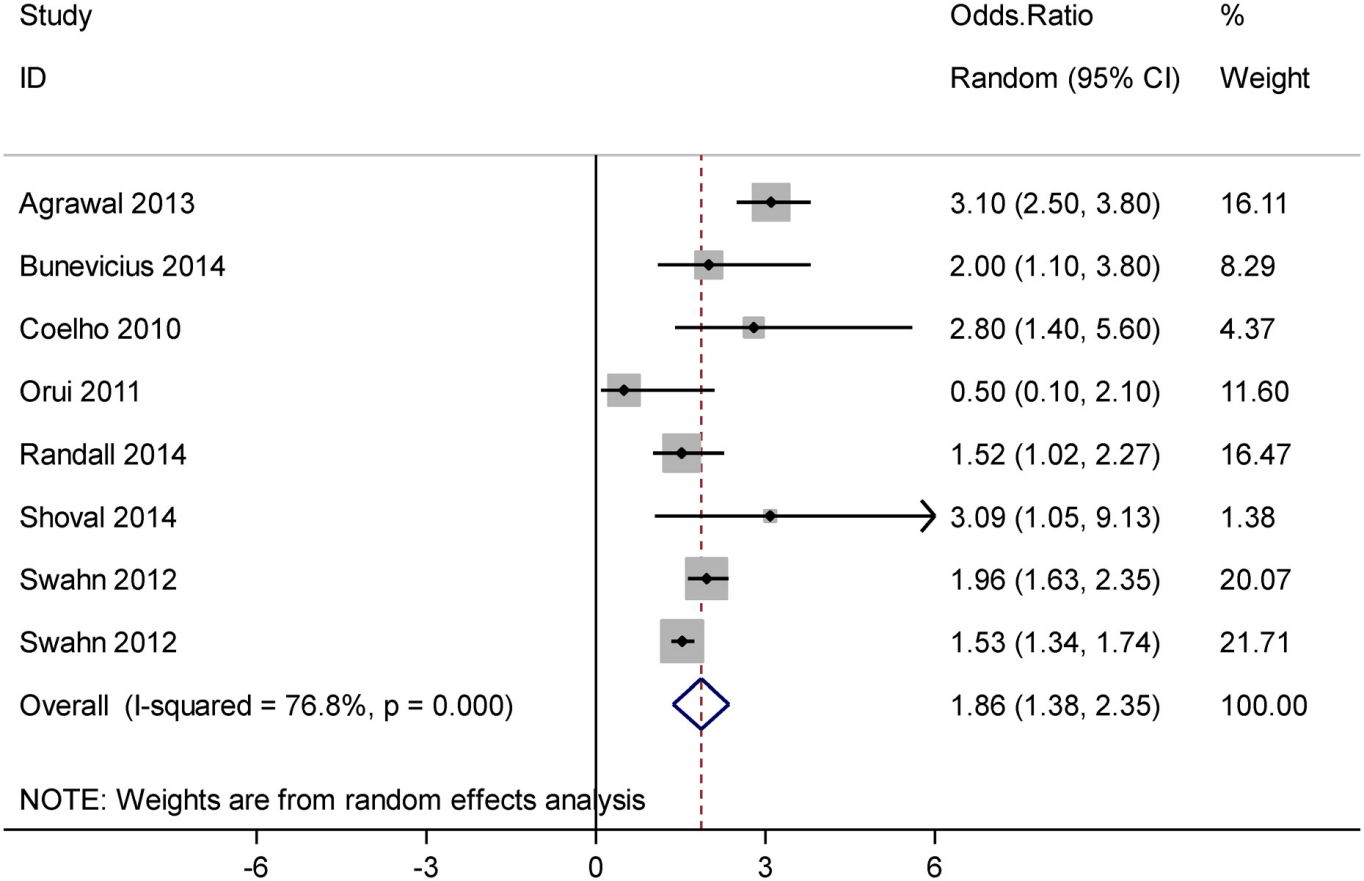
Forest plot of the association between alcohol use disorder and suicide ideation.

Fig 2 shows the results of the meta-analysis addressing the association between AUD and suicidal ideation. Based on this forest plot, AUD was significantly associated with suicidal ideation, OR = 1.86 (95% CI: 1.38, 2.35).

**Fig 3:**
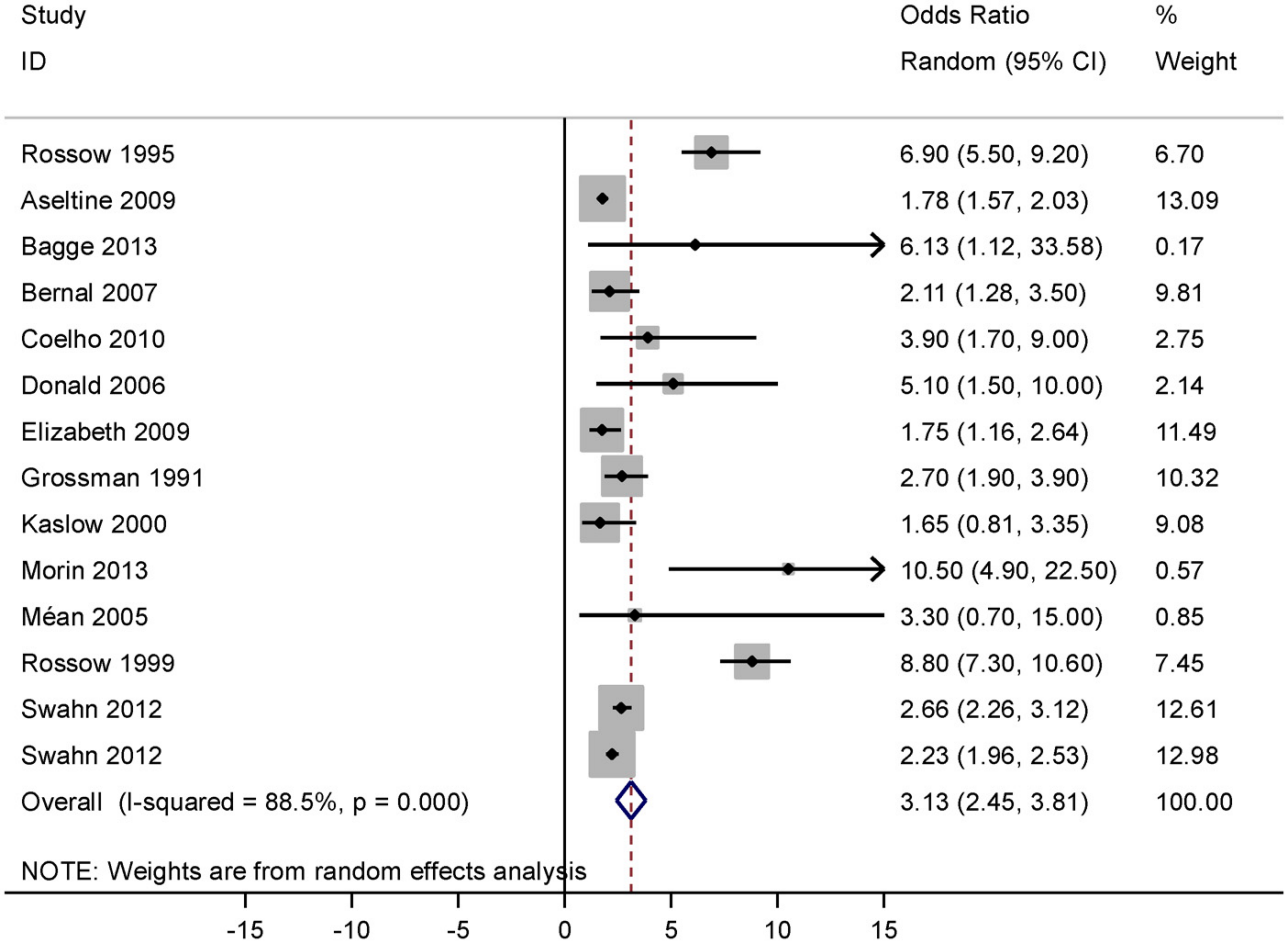
Forest plot of the association between alcohol use disorder and suicide attempt.

Fig 3 gives the forest plot of the association between AUD and suicide attempt. According to this forest plot, AUD was strongly associated with suicide attempt, OR = 3.13 (95% CI: 2.45, 3.81). There was an extreme value (outlier) [28] among the studies with OR = 18.0 (95% CI: 2.75, 118) that was excluded from the meta-analysis.

**Fig 4:**
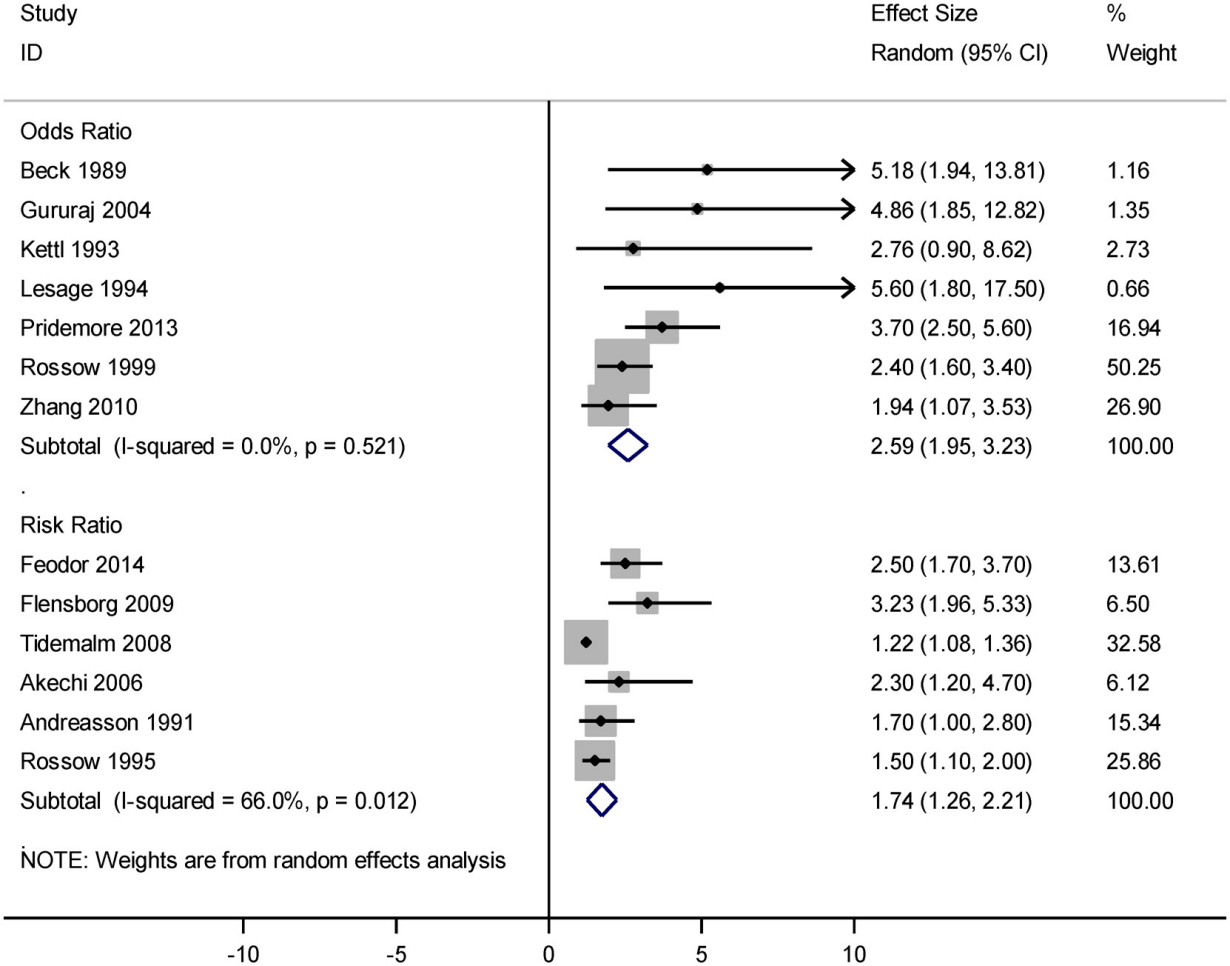
Forest plot of the association between alcohol use disorder and completed suicide.

Fig 4 represents the forest plot of the association between AUD and completed suicide. Based on this forest plot, AUD was significantly associated with completed suicide, OR = 2.59 (95% CI: 1.95, 3.23) and RR = 1.74 (95% CI: 1.26, 2.21). There was an extreme value [41] with OR = 0.541 (95% CI: 0.30, 0.97) that was excluded from the meta-analysis.

### Heterogeneity and publication bias

Heterogeneity was explored using Q-test and the quantity of heterogeneity was measured by the I^2^ statistic (Figs 2–4). Fig 2. shows a substantial heterogeneity (I^2^ = 76.8%, P<0.001) among studies addressing the association between AUD and suicidal ideation. Fig 3. indicates a considerable heterogeneity (I^2^ = 88.5%, P<0.001) among studies addressing the association between AUD and suicide attempt. As shown in Fig 4, there was no evidence of heterogeneity (I^2^ = 0.0%, P = 0.521) across studies reporting OR estimates of AUD related completed suicide, but there was a moderate heterogeneity (I^2^ = 66.0%, P = 0.012) among the studies reporting RR.

**Fig 5:**
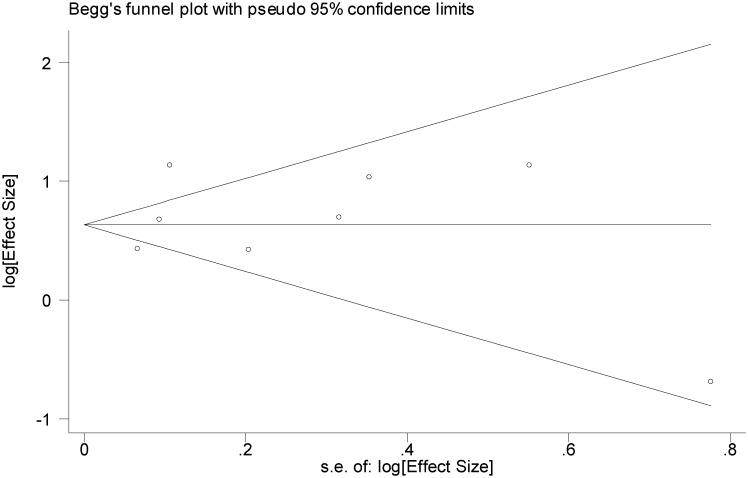
Funnel plot of included studies assessing the publication bias in studies addressing the association between alcohol use disorder and suicide ideation.

**Fig 6:**
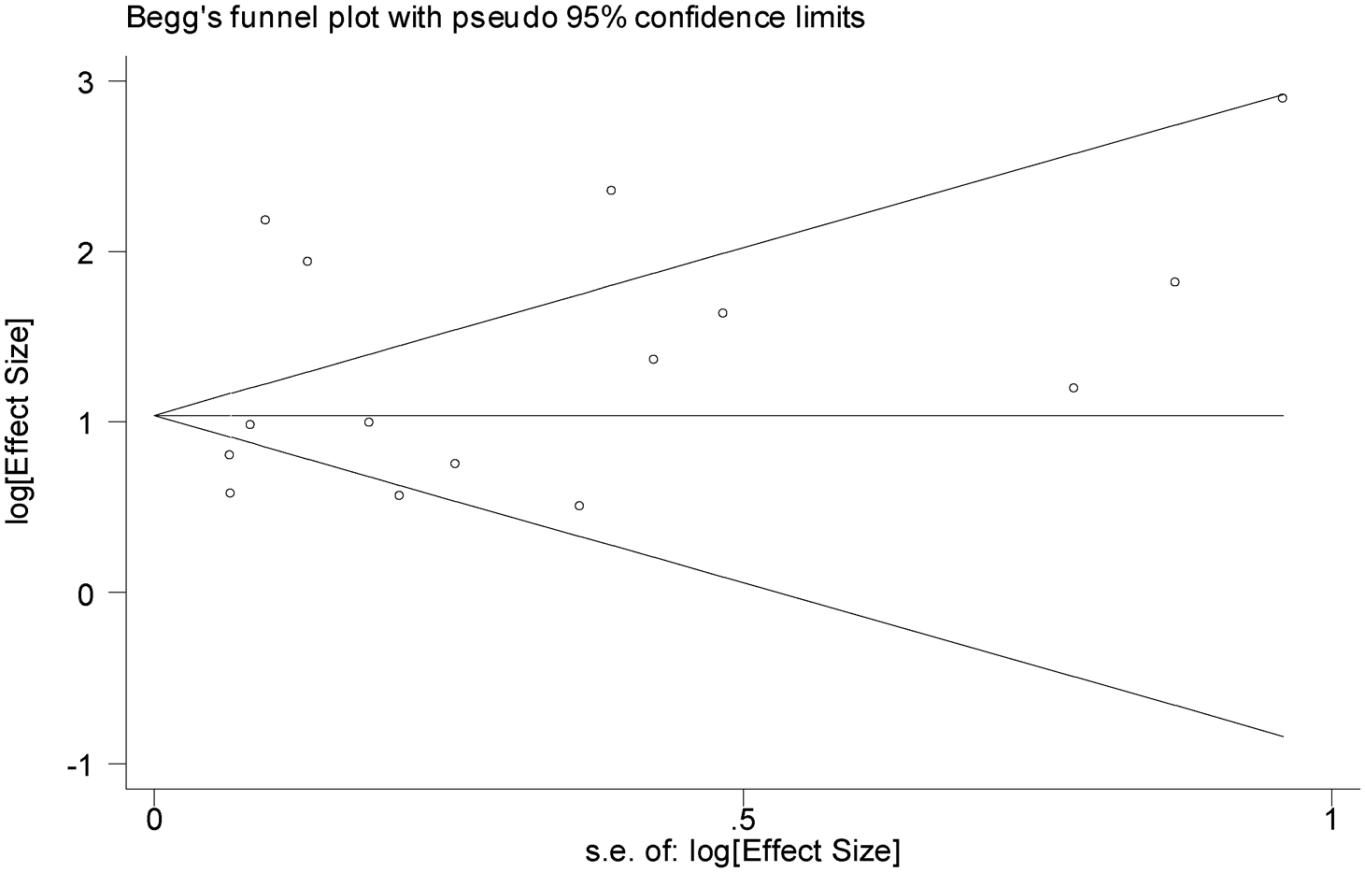
Funnel plot of included studies assessing the publication bias in studies addressing the association between alcohol use disorder and suicide attempt.

**Fig 7:**
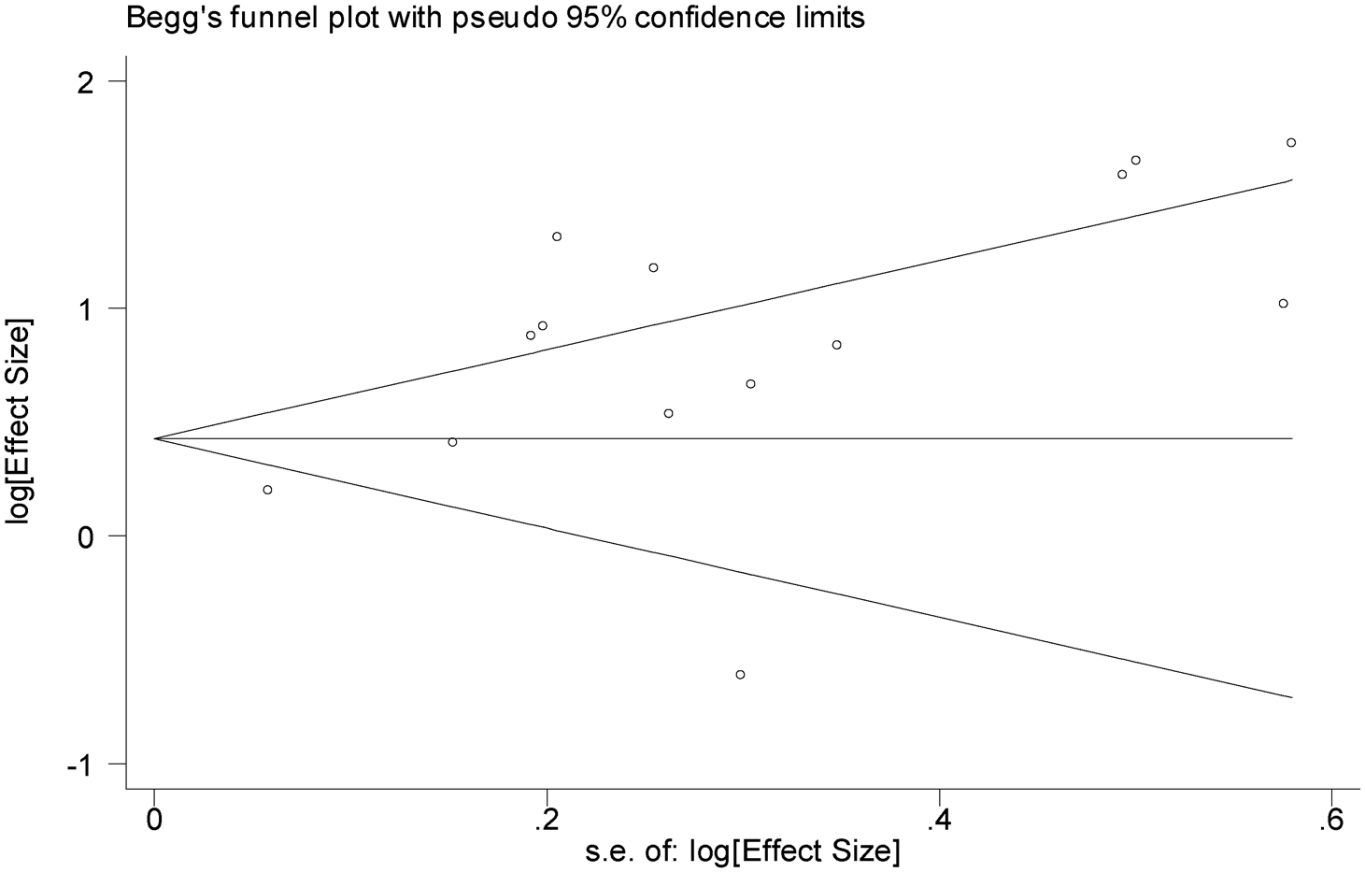
Funnel plot of included studies assessing the publication bias in studies addressing the association between alcohol use disorder and completed suicide.

We explored the possibility of publication bias using the funnel plot (Figs 5–7) as well as the Egger's and Begg's statistical tests. Based on Figs 5 and 6, the studies are scattered nearly symmetrically on both sides of the horizontal line indicating no evidence of publication bias in the studies reflecting the association between AUD and suicidal ideation and suicide attempt. The results of Egger's and Begg's tests confirmed this issue and revealed no evidence of publication bias among the studies addressing the association between AUD and suicidal ideation (P = 0.740 and P = 0.805) and suicide attempt (P = 0.363 and P = 0.125), respectively, As shown in Fig 7, studies are scattered asymmetrically on both sides of the horizontal line reflecting evidence of publication bias among the studies addressing the association between AUD and completed suicide. The Egger's test was statistically significant (P = 0.008), but the Begg's test was not (P = 0.477).

### Moderator analysis

In order to explore the sources of heterogeneity, we performed meta-regression analysis considering mean age, gender (percent of men), adjusted/unadjusted effect estimates, and a high/low risk of bias as covariates (Table 2). Multivariate meta-regression indicates the impact of moderator variables on study effect size. According to the results of meta-regression analysis, none of the covariates had a significant effect on the observed heterogeneity. The effects of covariates in the meta-regression are presented based on a logarithmic scale, so that it is possible to estimate the risk of suicidal ideation, attempted suicide, and completed suicide based on different scenarios of moderator variables.

**Table 2:** Analysis of meta-regression exploring sources of heterogeneity considering mean age, sex, adjusted versus unadjusted effect estimates, and studies with a high risk of bias versus those with a low risk of bias as covariates based on logarithmic scale.

Variables	Coefficient	SE	95% CI	*P* value
**Suicide ideation**				
Mean age (yr)	-0.043	0.032	-0.182, 0.096	0.313
Gender (% of men)	0.783	1.822	-7.058, 8.625	0.709
Adjustment (0 = unadjusted; 1 = adjusted)	-0.115	0.995	-4.396, 4.165	0.918
Risk of bias (0 = high risk; 1 = low risk)	1.263	1.011	-3.089, 5.616	0.338
Constant	0.804	0.963	-3.341, 4.949	0.482
**Attempted suicide**				
Mean age (yr)	0.015	0.010	-0.009, 0.040	0.195
Gender (% of men)	0.836	0.633	-0.623, 2.297	0.223
Adjustment (0 = unadjusted; 1 = adjusted)	0.061	0.345	-0.736, 0.859	0.863
Risk of bias (0 = high risk; 1 = low risk)	0.345	0.482	-0.766, 1.457	0.494
Constant	0.159	0.469	-0.923, 1.241	0.743
**Completed suicide**				
Mean age (yr)	-0.017	0.017	-0.057, 0.022	0.357
Gender (% of men)	-0.072	0.857	-2.011, 1.867	0.935
Adjustment (0 = unadjusted; 1 = adjusted)	-0.015	0.394	-0.908, 0.876	0.969
Risk of bias (0 = high risk; 1 = low risk)	0.591	0.460	-0.450, 1.634	0.231
Constant	1.126	1.174	-1.529, 3.782	0.363

## Discussion

This meta-analysis revealed that AUD was significantly associated with an increased risk of suicidal ideation, suicide attempt, and completed suicide. Alcohol and psychiatric disorder have a complicated relationship. Alcohol drinking can have negative effects on mental health, causing psychiatric disorders and increasing the risk of suicide [54]. Countries that have higher rates of alcohol use generally also have higher rates of suicide [55]. Furthermore, current evidence indicates an association between alcohol dependence and impulsive suicide attempts [56,57]. In addition, there is a close link between alcohol abuse and depression and it is often difficult to determine which of the two is the main leading condition [58]. Pompili et al have suggested that people with alcohol disorder should be screened for suicidality as well as psychiatric disorders [59].

Smith et al [12] conducted a meta-analysis in 1999 and reported that the overall percentage of the blood alcohol concentration was significantly higher in suicide cases. However, the magnitude of the suicide risk was not evaluated. Fazel et al [13] conducted a meta-analysis to examine the risk factors associated with suicide in prisoners. They reported that risk of completed suicide increases 3-fold in prisoners with a history of alcohol use. This effect estimate is greater than our estimate. This inconsistency between the results is acceptable because, several studies have indicated that suicide rates in prisoners are 5 to 10 times higher than the general population [60,61].

As shown in Fig 4, the summary measure obtained from OR, estimating the risk of completed suicide, was greater than that obtained from RR. The reason is straightforward because OR inherently tends to exaggerate the magnitude of the association [62].

There was a significant heterogeneity between the included studies (small P value of Q-test and large I^2^ statistic). The results of the statistical tests assessing heterogeneity should be interpreted with caution. When the sample size is small or the number of studies is limited, the Q-test has low statistical power. On the other hand, when the sample size or the number of the included studies is large, the test has high power in detecting a small amount of heterogeneity that may be clinically unimportant [18]. Therefore, a part of observed heterogeneity can be attributed to the number of studies (31 studies) included in the meta-analysis and the large sample size (involving 420,732 participants). However, another part of observed heterogeneity can be attributed to the discrepancies across the studies. The OR estimates of suicidal ideation were reported from 0.5 to 3.10 and that of suicide attempt from 1.65 to 10.50. The source of observed heterogeneity was explored using a meta-regression analysis considering mean age, sex, adjusted/unadjusted effect estimates, and methodological quality of the included studies as covariates. However, none of these covariates had a significant effect on the observed heterogeneity. One reason that may explain this heterogeneity is that individual studies come from different settings with different populations, sample sizes and methodological quality.

There was an outlier (OR = 18.0) [28] among the included studies addressing the association between AUD and suicide attempt. This cohort study was a report of research on suicide attempt based on an analysis of data from the Epidemiologic Catchment Area surveys, enrolling 13,673 participants, in the United States, in the early 1980s. This study reported the association between active alcoholism and suicide attempt using multiple conditional logistic regression analysis. The full text of this study was not accessible for further evaluation of the study population and its associated eligibility criteria. Thus, the reason of this extreme value remained unclear.

There were some limitations in this meta-analysis as follows. First, wherever possible, we used the full adjusted forms of RR and OR controlling for factors such as age, gender, race, mental disorder, drug abuse, smoking, marital status, body mass index, educational level, employment status, income, and living alone. However, the confounding effect was not completely ruled out because some studies reported crude forms of RR or OR estimates. This issue may lead to overestimation of the overall measures of association. Second, there were 12 studies (mostly old studies) that seemed potentially eligible for inclusion in this meta-analysis, but their full texts were not accessible. We requested the relevant institutes to find the full texts for us, but they could not. We tried to contact the corresponding authors to send us the full texts, but the authors did not respond. This issue may raise the possibility of selection bias.

Despite the above limitations, the current meta-analysis could efficiently estimate the association between AUD and suicide. Furthermore, a wide search strategy was developed in order to increase the sensitivity of the search to include as many studies as possible. Our study included all types of observational studies irrespective of age, country, race, publication date, and language. We screened 8548 retrieved references and included 31 eligible studies in the meta-analysis involving 420,732 participants. Thus, the evidence was sufficient to make a robust conclusion regarding the objective of the study for estimating the association between AUD and suicide.

We can have high confidence based on the current evidence that AUD increases the risk of suicide. Therefore, further research is very unlikely to change our confidence in the estimate of effect. This finding supports the alcohol cessation programs to reduce alcohol use among the general population. However, there is insufficient evidence in regard to the dose-response relationship between alcohol drinking and risk of suicide. Further investigation based on observational studies are needed to expect the dose-response pattern of alcohol-related suicide.

## Conclusion

This meta-analysis measured the association between AUD and suicide. Based on current evidence, AUD significantly increases the risk suicidal ideation, suicide attempt, and completed suicide. Therefore, AUD can be considered an important predictor of suicide and a great source of premature death.

## Supporting Information

S1 PRISMA Checklist (DOC)
