# Correction: Alcohol-Related Risk of Suicidal Ideation, Suicide Attempt, and Completed Suicide: A Meta-Analysis

**DOI:** 10.1371/journal.pone.0241874

**Published:** 2020-10-29

**Authors:** Nahid Darvishi, Mehran Farhadi, Tahereh Haghtalab, Jalal Poorolajal

After publication of this article [1], concerns were raised about the inclusion of the study by Rossow and Amundsen [2] in the analysis shown in Fig 3, as this study reported estimates of the association between alcohol use disorder (AUD) and completed suicides but did not report on the association between AUD and suicide attempts. The authors apologize for this error and present here an updated version of Fig 3 in which this study is excluded. In the updated analysis, the summary measure of the effect of AUD on suicide attempt was 2.79 (95% CI: 2.16, 3.43), compared to 3.13 (95% CI: 2.45, 3.81) in the original analysis [1]. However, the association between AUD and suicide attempt remained significant and strong (P<0.001). The heterogeneity across included studies, based on I^2^ statistics, reduced from 88.5% in [1] to 86.3% in the reanalysis. The funnel plot analysis for attempted suicide (Fig 6) was also corrected accordingly, excluding the Rossow 1995 article from the analysis. Please see the updated Figs 3 and 6 here.

**Fig 3:**
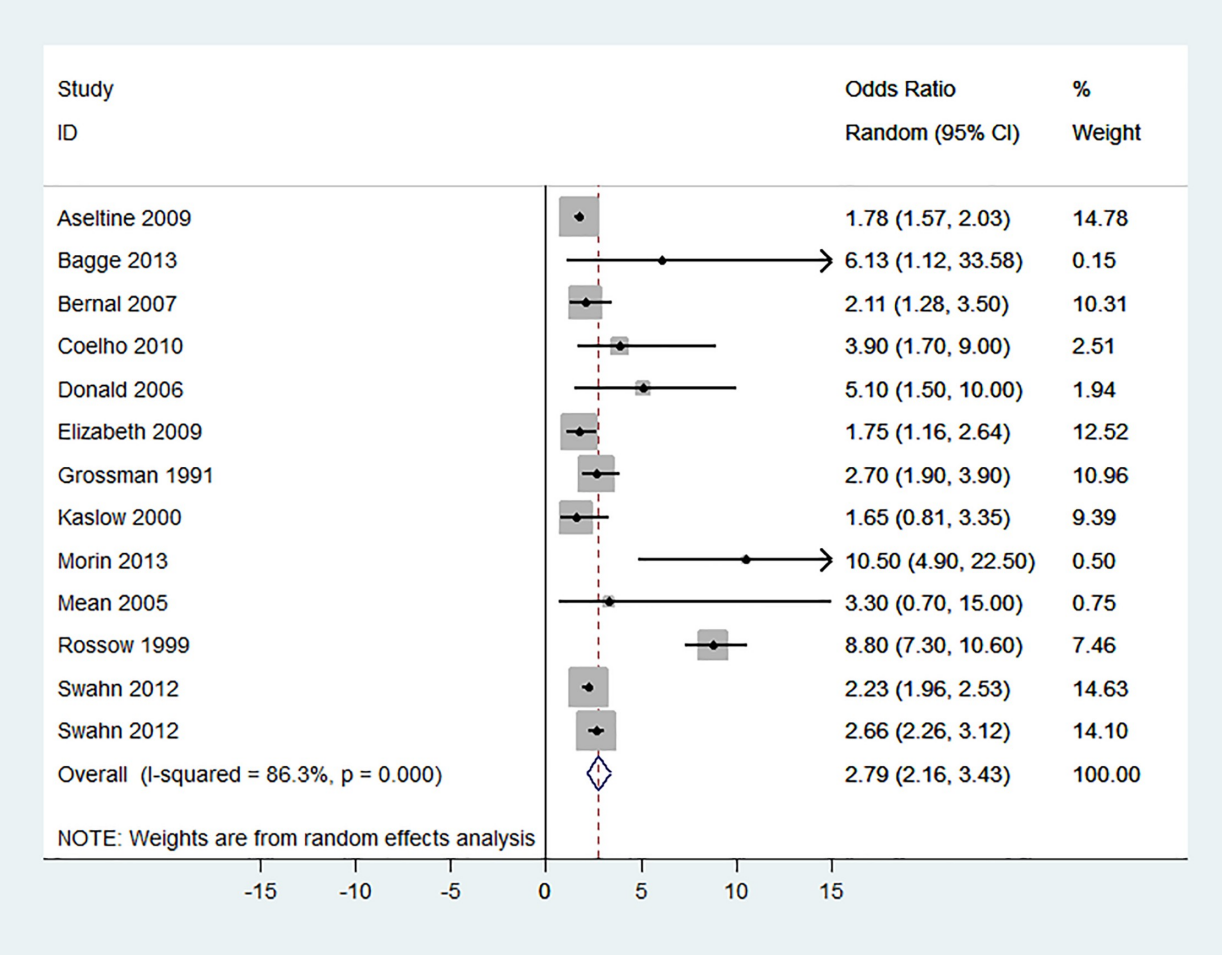
Forest plot of the association between alcohol use disorder and suicide attempt.

**Fig 6:**
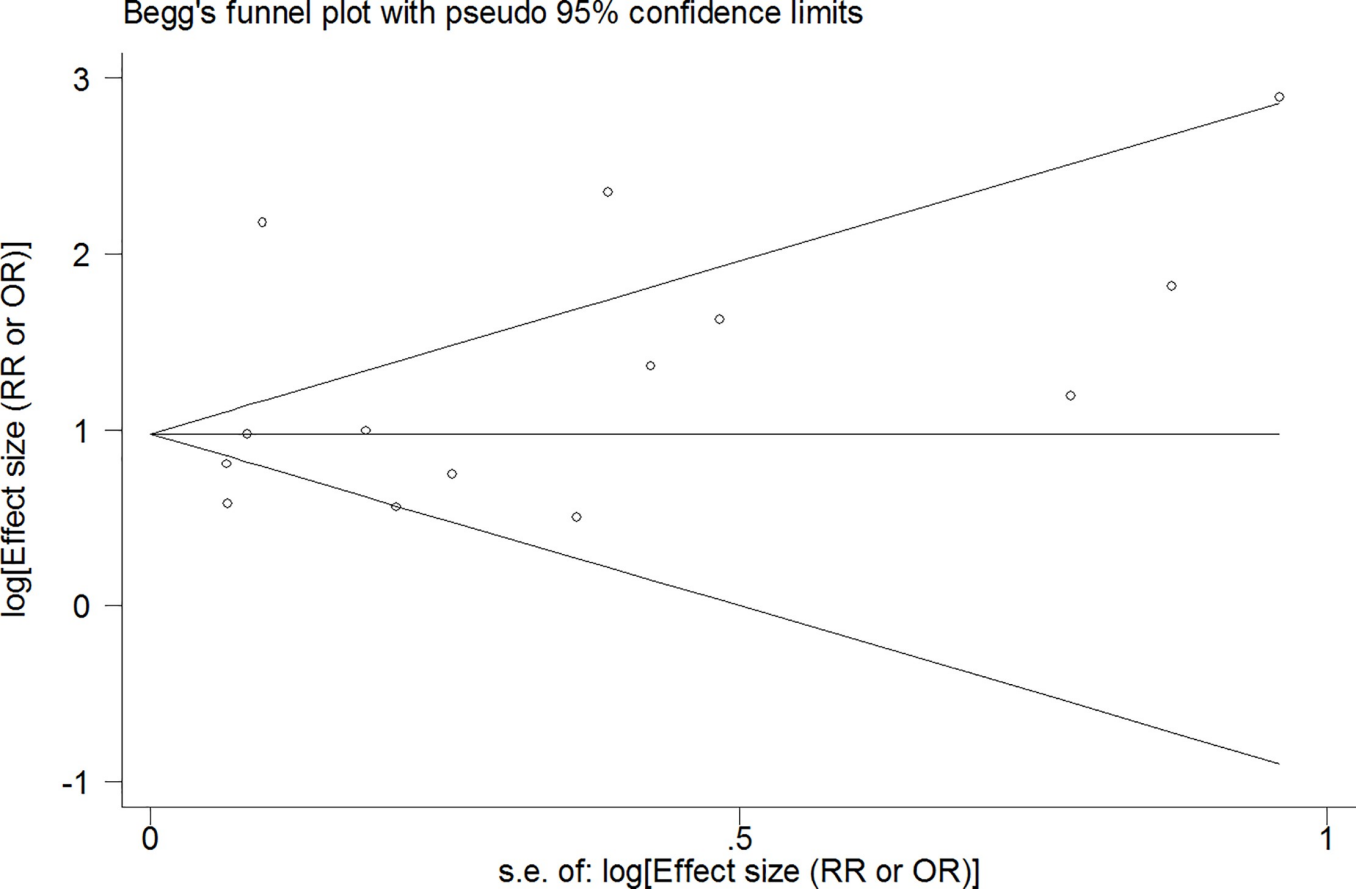
Funnel plot of included studies assessing the publication bias in studies addressing the association between alcohol use disorder and suicide attempt.

Questions were raised about the RR value used for the Rossow (1995) article, which is reported as 6.90 in Fig 3 and as 1.50 in Fig 4 in [1]. The authors clarify that the correct RR value (1.50) was used in the analysis reported in Fig 4. Whereas the 6.90 figure is reported in Rossow (1995) in Table 1, the text of that article reports that the odds of suicide among those who died was 1.50 higher among alcohol abusers compared to non-abusers (p. 688 of [2]).

**Table 1:** Summary of study results.

		Age (yr)							Newcastle Ottawa Score		
1^st^ author	Country	Mean	Range	Gender	Population	Study	Estimate	Sample	Sel	Com	E/O
Agrawal 2013	USA	15.9	18–27	Female	General	Cross-sectional	Crude	3,787	****	*	**
Akechi 2006	Japan	49.5	40–69	Male	General	Cohort	Adjusted	43,383	****	**	***
Andreasson 1991	Sweden	-	18–21	Male	General	Cohort	Adjusted	49,464	***	**	**
Aseltine 2009	USA	-	11–19	Both	General	Cross-sectional	Adjusted	32,217	**	**	**
Bagge 2013	USA	36.7	18–64	Both	General	Case-Control	Adjusted	192	****	**	**
Beck 1989	USA	29.9	-	Both	General	Case-Control	Adjusted	413	***	**	**
Bernal 2007	Europe	47.0	18+	Both	General	Cross-sectional	Adjusted	21,425	***	**	**
Bunevicius 2014	Lithuania	50.0	18–89	Both	General	Cross-sectional	Adjusted	998	***	**	**
Coelho 2010	Brazil	-	18+	Both	General	Cross-sectional	Adjusted	1,464	**	**	**
Donald 2006	Australia	-	18–24	Both	General	Case-Control	Adjusted	380	****	**	**
Schilling 2009	USA	-	13–18	Both	General	Cross-sectional	Crude	31,953	**	*	*
Feodor 2014	Denmark	-	16+	Both	General	Cohort	Adjusted	32,010	****	**	***
Flensborg 2009	Denmark	-	20–93	Both	General	Cohort	Adjusted	18,146	****	**	***
Grossman 1991	USA	14.4	-	Both	General	Cross-sectional	Adjusted	6,637	***	**	***
Gururaj 2004	India	-	15–60	Both	General	Case-Control	Crude	538	****	*	**
Kaslow 2000	USA	30.8	18–64	Female	General	Case-Control	Crude	285	***	*	**
Kettl 1993	Alaska	30.8	-	Both	General	Case-Control	Crude	66	**	*	***
Lesage 1994	Canada	-	18–35	Male	General	Case-Control	Crude	150	****	*	***
Méan 2005	Switzerland	18.9	16–21	Both	General	Cohort	Adjusted	148	***	**	*
Morin 2013	Sweden	80.0	70–91	Both	General	Case-Control	Adjusted	515	***	**	**
Orui 2011	Japan	54.0	-	Both	General	Cross-sectional	Adjusted	770	***	**	**
Petronis 1990	USA	-	-	Both	General	Cohort	Adjusted	13,673	FTU	FTU	FTU
Pridemore 2013	Russia	-	25–54	Male	General	Case-Control	Adjusted	1,640	****	**	**
Randall 2014	Benin	-	12–16	Both	General	Cross-sectional	Adjusted	2,690	**	**	**
Rossow 1999	Sweden	-	25–44	Male	General	Cohort	Adjusted	46,490	****	**	***
Shoval 2014	Israel	-	21–45	Both	General	Cross-sectional	Adjusted	1,237	***	**	**
Swahn 2012	France	-	11–19	Both	General	Cross-sectional	Adjusted	13,187	**	**	**
Swahn 2012	USA	-	11–19	Both	General	Cross-sectional	Adjusted	15,136	**	**	**
Tidemalm 2008	Sweden	37.7	-	Both	General	Cohort	Adjusted	39,685	****	**	***
Zhang 2010	China	-	-	Male	General	Cross-sectional	Adjusted	454	**	**	**
Zonda 2006	Hungary	52.0	-	Both	General	Case-Control	Crude	200	***	*	**

**Sel**: Selection; **Com**: Comparability; **E/O**: Exposure/Outcome; **FTU**: Full text unavailable

**Adjusted** means controlled for one or more of the following factors: age, gender, race, mental disorder, drug abuse, smoking, marital status, body mass index, educational level, employment status, income, living alone

Concerns were also raised about the heterogeneity in outcome measures analyzed, which included early alcohol initiation, “any heavy drinking”, and AUD. For example, it was noted that [3] reported on early alcohol initiation and “any heavy drinking” (rather than AUD) as an exposure measure, and reported outcomes of self-reported suicidal behavior rather than a suicide outcome based on hospital records. The authors clarified that the upper part of Table 3 in [3] reported the effect of age of alcohol initiation as exposure measure on a suicide attempt. The lower part of the table reported the effect of various behavioral risk factors on the suicide attempt, including "any heavy drinking" which was used as a proxy for AUD. As mentioned in the Methods, the analysis of the association between AUD and suicide based on observational studies included data obtained using multiple study designs (cohort, case-control, and cross-sectional studies). However, the authors differentiated between the types of suicide (ideation, attempt, and complete) and the measure used to report effect estimates (odds ratio and risk/hazard ratio), as shown in Fig 4. In light of the heterogeneity between studies, the authors performed a meta-analysis using random-effects model, analyzed the heterogeneity across the included studies using I^2^ statistics, and performed meta-regression to explore sources of heterogeneity (Table 2 in [1]).

The authors provide here an updated version of Table 1 in which participant age groups are reported consistently as the age range and/or mean age, per data available in the primary literature.

In addition, the Data Availability Statement for this article [1] is incorrect. The primary data are not provided in the published paper and its Supporting Information files. The authors provide the full list of excluded articles (with reasons for exclusion) and the table of all data extracted from the primary literature and used in the meta-analysis as Supporting Information in this Correction.

Finally, there are errors in the References for this article [1].

The author names are not listed correctly for Reference 47. Note, in Fig 3 and Table 1 in [1] this reference is cited as Elizabeth 2009. The correct reference is:

Schilling EA, Aseltine RH Jr, Glanovsky JL, James A, Jacobs D. Adolescent alcohol use, suicidal ideation, and suicide attempts. J Adolesc Health. 2009;44: 335–341. pmid:19306791.

Additionally, the publication year is incorrectly listed as 2014 for Reference 52 in [1]. The correct reference is:

Swahn MH, Bossarte RM, Choquet M, Hassler C, Falissard B, Chau N. Early substance use initiation and suicide ideation and attempts among students in France and the United States. Int J Public Health. 2012;57: 95–105.

## Supporting information

S1 File Characteristics of the excluded studies. (DOC)

S2 File Dataset underlying the study. (XLSX)
