# Expression of Concern: Alcohol-Related Risk of Suicidal Ideation, Suicide Attempt, and Completed Suicide: A Meta-Analysis

**DOI:** 10.1371/journal.pone.0279589

**Published:** 2022-12-19

**Authors:** The PLOS ONE Editors

Following the publication of this article and Correction [1, 2], additional concerns were raised regarding the presentation of results and the systematic review methodology.

Specifically, the X-axis scale and the Y-axis midline indicating “no effect” are not presented correctly in the forest plots shown in Figs 4–4 in [1] and Fig 3 in [2].

A member of the Editorial Board evaluated this article and expressed concern about how the systematic review was conducted due to the following issues which raise concern about potential bias in the results:

- The results of two studies [3, 4] were excluded from analyses as the authors determined that the results were extreme values.
- 12 studies that were potentially eligible for inclusion in the meta-analysis were not evaluated for inclusion because the full text was not available.
- At least one potentially relevant study [5] was not identified during the systematic review and was therefore not assessed for inclusion in the meta-analysis.

The corresponding author acknowledged the errors in the presentation of Figs 2–4 and provided updated versions with the X-axes and midline presented correctly (Figs 2–4 with this notice). They also provided re-analysis of the results in Figs 3 and 4 with [3] and [4] included, respectively. The estimates did not change significantly, and the Editorial Board member agreed that these issues have been addressed.

**Fig 2:**
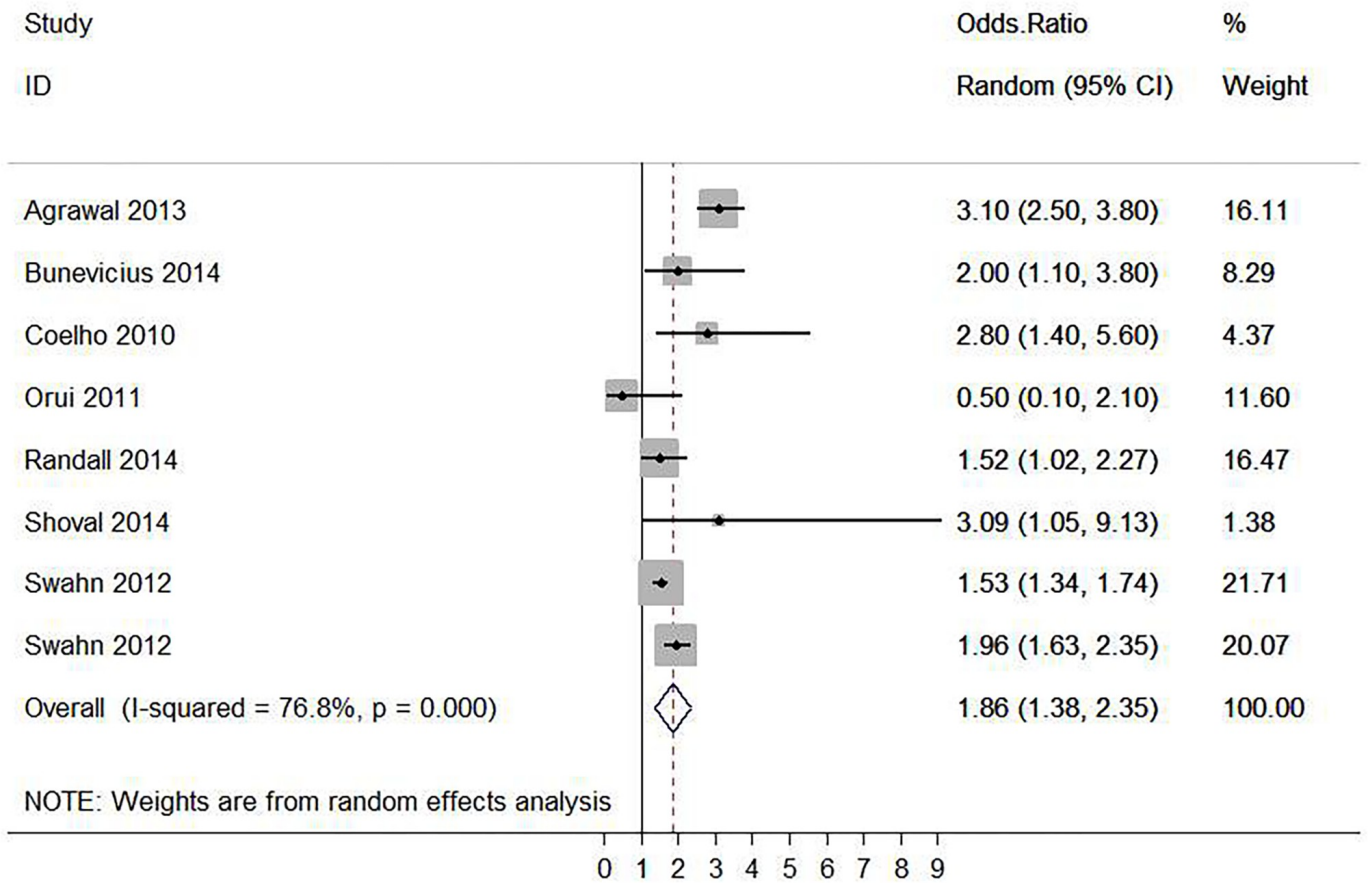
Forest plot of the association between alcohol use disorder and suicide ideation.

**Fig 3:**
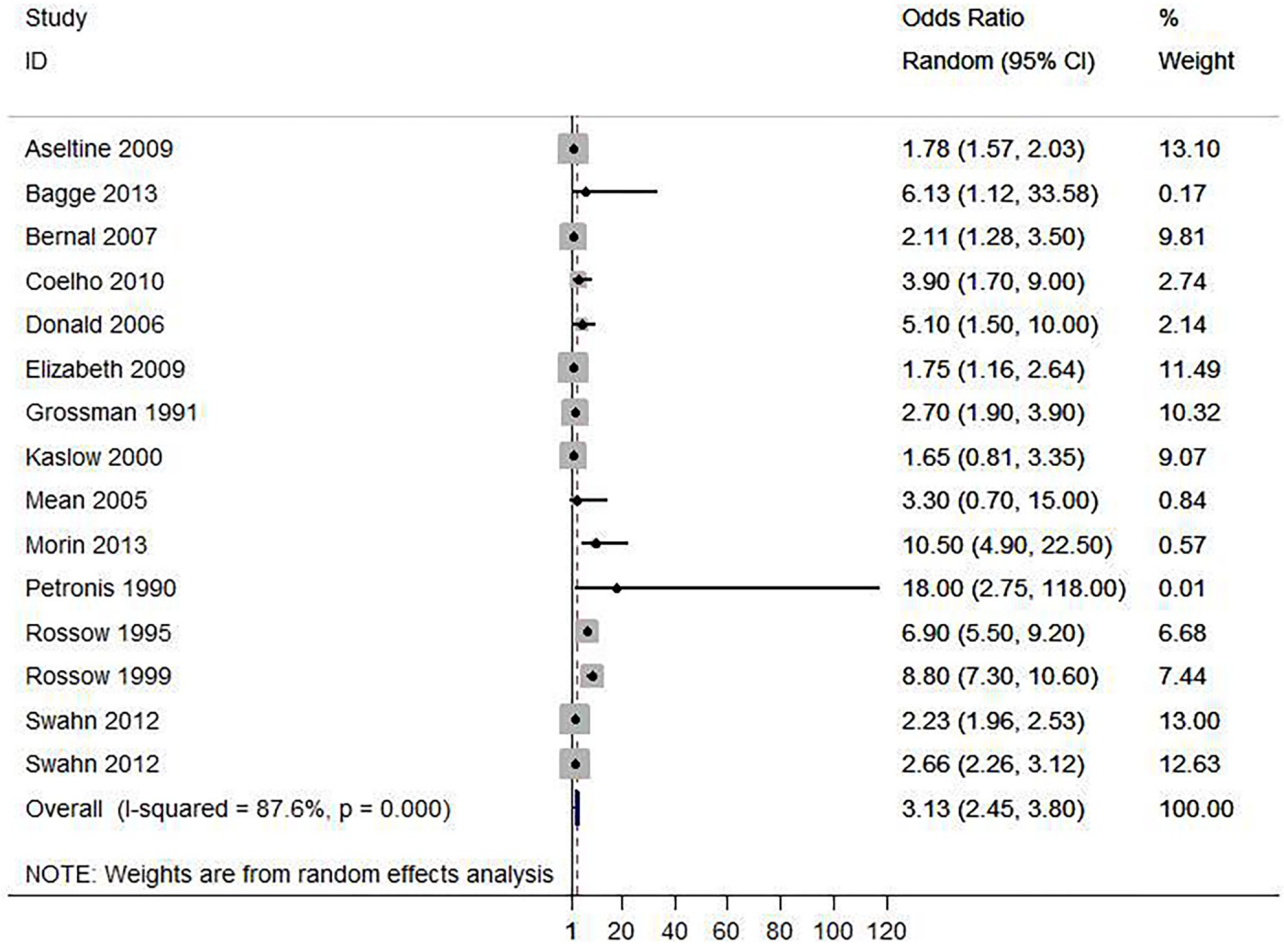
Forest plot of the association between alcohol use disorder and suicide attempt.

**Fig 4:**
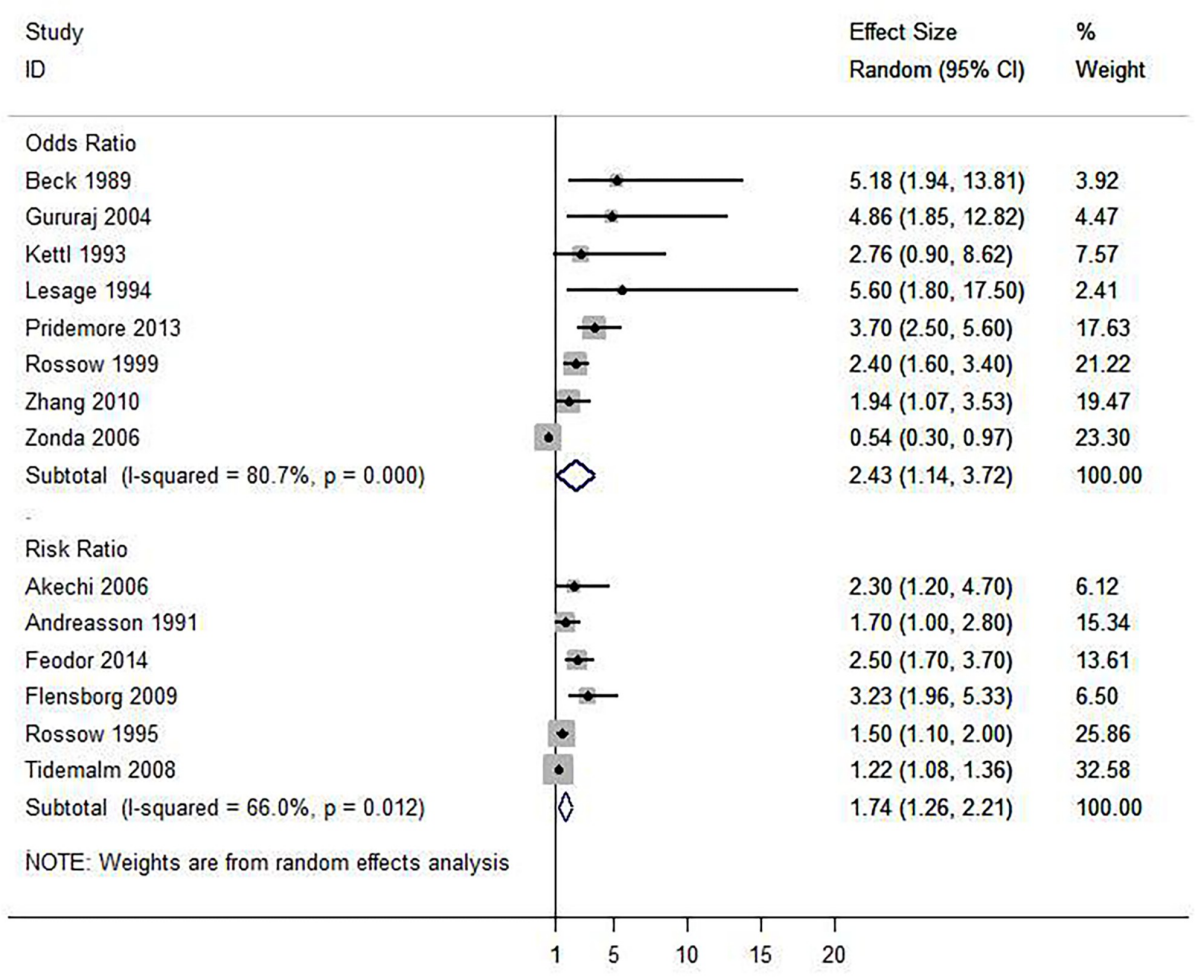
Forest plot of the association between alcohol use disorder and completed suicide.

The corresponding author and editors note that the potential limitation of not including 12 studies for which the full text could not be retrieved was acknowledged in the discussion section of [1].

The editors remain concerned about the rigour of the systematic review methodology and that potentially relevant studies were not evaluated for inclusion.

The *PLOS ONE* Editors issue this Expression of Concern to notify readers of the above concerns and to relay the updated figures provided by the corresponding author.
