# Supplementary material for: Correction: Alcohol-Related Risk of Suicidal Ideation, Suicide Attempt, and Completed Suicide: A Meta-Analysis
Source: PLoS One. 2020 Oct 29;15(10):e0241874. doi: 10.1371/journal.pone.0241874 (PMC7595431; doi:10.1371/journal.pone.0241874)
Supplement: S1 File — (DOC) [file pone.0241874.s001.doc]

**Appendix 2**: Characteristics of the excluded studies

| **Row** | **Study** | **Reason for exclusion** |
| --- | --- | --- |
|  | Agarwal 2010 | No comparison group. |
|  | Allebeck 1987 | The association between alcohol and suicide was investigated among patients with mental disorder. |
|  | Allebeck 1990 | The association between alcohol and suicide was investigated among patients with schizophrenia. |
|  | Allen 2011 | A review article. |
|  | Allen 2014 | No association between AUD and suicide was reported. |
|  | Al-Sharqi 2012 | No comparison group |
|  | Alvarado-Esquivel 2014 | The association between alcohol and suicide was investigated among patients with mental disorder. |
|  | Bagge 2013 | A text book. |
|  | Batchelor 1954 | The sociological caracteristics of alcohol use on suicide was investigated. |
|  | Beck 1989 | The effect of alcohol use just before index attempt on suicide was evaluated. |
|  | Berglund 1984 | There was no control group. |
|  | Berglund 1987 | No association between alcohol and suicide was reported. |
|  | Berman 2013 | The suicide risk and local option status under state law about alcohol was addressed. |
|  | Berman 2014 | The effectiveness of local alcohol control was evaluated. |
|  | Binder 2001 | No association between alcohol and suicide was reported. |
|  | Boenisch 2010 | No association between AUD and suicide was reported. |
|  | Borges 2004 | The effect alcohol use within 6 hours of suicide attempt rather than alcohol abuse/dependence. |
|  | Borges 2010 | A review article rather than original article. |
|  | Branas 2011 | The effect of acute alcohol consumption on suicide rather than AUD was investigated. |
|  | Burch 1994 | No association between AUD and suicide was reported. |
|  | Buri 2009 | No association between AUD and suicide was reported. |
|  | Caetano 2013 | The effect of acute alcohol intoxication on suicide was investigated. |
|  | Canapary 2002 | No association between AUD and suicide was reported. |
|  | Carpenter 2004 | No association between AUD and suicide was reported. |
|  | Chachamovich 2012 | The association between alcohol-related suicides with other factors were reported |
|  | Chan 2013 | Relationship between alcohol and deliberate self-harm rather than suicide |
|  | Chaveepojnk 2011 | No association between alcohol and suicide was reported. |
|  | Cheng 2000 | Combine effect of alcohol and drug abuse on suicide was investigated. |
|  | Cheryl 2004 | A review article rather than original article. |
|  | Chojnicka 2013 | The association between gens, which modulate ethanol consumption, and suicide |
|  | Chun 2013 | Self-inflicted intentional injury rather than suicide attempt was reported |
|  | Conner 2001 | The association between suicide and violent behavior rather than alcohol was reported. |
|  | Conner 2001 | The interaction of violence and alcohol misuse and suicide was investigated. |
|  | Conner 2003 | The interaction between alcohol dependence and other factors and suicide was investigated. |
|  | Cottler 2005 | The association between alcohol and suicide was investigated among drug abusers |
|  | Davis 1993 | The comparison of the duration of time among those who had committed suicide. |
|  | Dawson 1997 | Suicide attempt and ideation were evaluated simultaneously. |
|  | Dearden 2005 | The definition of alcohol abuse/dependence is unclear. |
|  | Delavenne 2011 | No association between alcohol and suicide was reported. |
|  | Dumais 2005 | The association between alcohol and suicide was investigated among patients with mental disorder. |
|  | Dutta 2007 | The association between alcohol and suicide was investigated among patients with mental disorder. |
|  | Dvorak 2013 | No association between AUD and suicide was reported. |
|  | Fazel 2008 | A meta-analysis rather than original article. |
|  | Frances 1987 | A text book. |
|  | Frederic 2004 | No association between AUD and suicide was reported. |
|  | Fudalej 2009 | No association between AUD and suicide was reported. |
|  | Garlow 2007 | No association between alcohol and suicide was reported. |
|  | Gmel 1998 | No association between alcohol and suicide was reported. |
|  | Goldstein 2005 | No association between alcohol and suicide was reported. |
|  | Grøholt 1999 | Comparison of the suicide rate between males and females. |
|  | Grossman 1991 | Risk factors of suicide other than alcohol were evaluated |
|  | Hakko 2005 | Survival times rather than association between alcohol use and suicide was reported. |
|  | Hawton 1989 | No association between alcohol and suicide was reported. |
|  | Hawton 1993 | Alcohol and drug abuse related suicides were evaluated simultaneously. |
|  | Hawton 2013 | A systematic review. |
|  | Hesselbrock 1988 | No association between alcohol and suicide was reported. |
|  | Hlady 1988 | No association between alcohol and suicide was reported. |
|  | Ian Smith 1991 | No association between alcohol and suicide was reported. |
|  | Ilomaki 2007 | The association between alcohol and suicide was investigated among patients with mental disorder. |
|  | Innamoratia 2010 | No association between suicide and alcohol was reported. |
|  | Jacobson 1986 | Comparison of self-incineration with other type of suicide methods |
|  | James 1971 | A letter to the editor. |
|  | Jee 2011 | The definition of alcohol abuse/dependence is unclear. |
|  | Kaplan 2013 | A letter to the editor. |
|  | Kim 2012 | The association between alcohol and suicide was investigated among patients with depression. |
|  | Kittirattana~ 2014 | An effect of illicit drug use with alcohol use disorders on suicide rate was assessed. |
|  | Knop 1981 | No association between alcohol and suicide was reported. |
|  | Kokkevi 2012 | The definition of alcohol abuse/dependence is unclear. |
|  | Kõlves 2006 | Suicide rate was assessed between sever alcoholic versus moderate ones. |
|  | Lamis 2014 | No association between alcohol and suicide was reported. |
|  | Lejoyeuxa 2008 | No association between suicide and alcohol was reported. |
|  | Lester 1991a | A summary rather than an original article. |
|  | Lester 1991b | No association between alcohol and suicide was reported. |
|  | Lester 1993 | A summary rather than an original article. |
|  | Lisansky 1989 | The association between alcohol and suicide was investigated among patients with mental disorder. |
|  | Lundholm 2014 | No association between alcohol and suicide was reported. |
|  | Malone 1997 | Combine effect of alcohol and drug abuse on suicide was investigated. |
|  | Manza 2008 | No association between alcohol and suicide was reported. |
|  | Mash 2014 | The definition of alcohol abuse/dependence is unclear. |
|  | May 2002 | No association between alcohol and suicide was reported. |
|  | McLean 2012 | The association between alcohol and suicide was investigated among patients with mental disorder. |
|  | McNamara 2010a | Suicide ideation was not separated from attempted suicide cases. |
|  | McNamara 2010b | Suicide ideation was not separated from attempted suicide cases. |
|  | Merrill 1992 | No association between alcohol and suicide was reported. |
|  | Merrill 1999 | The association between alcohol and marijuana use was investigated. |
|  | Min 2012 | No association between alcohol and suicide was reported. |
|  | Moran 2012 | Relationship between alcohol and deliberate self-harm rather than suicide |
|  | Moustgaard 2014 | The effect of alcohol in non-tricyclic antidepressant users on suicide was investigated. |
|  | Mukamal 2007 | The definition of alcohol abuse/dependence is unclear. |
|  | Murphy 1992 | No association between alcohol and suicide was reported. |
|  | Nadorff 2014 | The effect of alcohol in the presence of insomnia on suicide was evaluated. |
|  | Nakaya 2007 | The definition of alcohol abuse/dependence is unclear. |
|  | Nemtsov 2003 | No association between alcohol and suicide was reported. |
|  | Norström 2012 | No association between alcohol and suicide was reported. |
|  | Oconnor 2014 | The definition of alcohol abuse/dependence is unclear. |
|  | Park 2008 | The definition of alcohol abuse/dependence is unclear. |
|  | Penttinen 2001 | The association between alcohol and suicide was investigated among patients with mental disorder. |
|  | Perez 2010 | The definition of alcohol abuse/dependence is unclear. |
|  | Pirkola 1999 | A nonpublished article. No association between suicide and alcohol was reported. |
|  | Pirkola 2004 | No association between alcohol and suicide was reported. |
|  | Powell 2001 | The definition of alcohol abuse/dependence is unclear. |
|  | Preuss 2002a | Assessing suicide among alcoholic people with or without mental disorder. |
|  | Preuss 2002b | The effect size of alcohol on suicide was not reported. |
|  | Reyes 2011 | The definition of alcohol abuse/dependence is unclear. |
|  | Riedi 2012 | Relationship between alcohol and deliberate self-harm rather than suicide |
|  | Rorsman 1982 | The author has passed away. |
|  | Rossow 1993 | No association between alcohol and suicide was reported. |
|  | Rossow 1996 | Irrelevant to alcohol and suicide association |
|  | Roy 2007 | No association between alcohol and suicide was reported. |
|  | Rudatsikira 2007 | The definition of alcohol abuse/dependence is unclear. |
|  | Schilling 2009 | Heavy episodic drinking rather than alcohol abuse was addressed. |
|  | Schneider 2009 | No association between alcohol and suicide was reported. |
|  | Schneider 2011 | Standard mortality ratio rather than odds/risk ratio was reported. |
|  | Séguin 2006 | No effect size was reported. |
|  | Shah 2009 | No association between alcohol and suicide was reported. |
|  | Sher 2005 | A letter to the editor. |
|  | Sher 2006 | Commentary rather than original article. |
|  | Sher 2009 | No association between alcohol and suicide was reported. |
|  | Silva 2014 | The definition of alcohol abuse/dependence is unclear. |
|  | Siziya 2012 | The association between alcohol and bullying rather than suicide was addressed. |
|  | Skarbo 2006 | The combination of the effect alcohol used and mental disorder on suicide |
|  | Smith 1999 | A meta-analysis rather than original article. |
|  | Swahn 2007 | The association between suicide and preteen initiation of alcohol use rather than alcohol abuse/dependence was investigated. |
|  | Swahn 2008 | The association between suicide and preteen initiation of alcohol use rather than alcohol abuse/dependence was investigated. |
|  | Swahn 2010a | Suicide ideation was not separated from attempted suicide cases. |
|  | Swahn 2010b | Early alcohol use initiation and suicide attempts were reported. |
|  | Swahn 2012a | Parental alcohol use rather than themselves |
|  | Swahn 2012b | Drunkenness rather than alcohol abuse/dependence and suicide was addressed. |
|  | Tamás | No effect size was reported. |
|  | Wagenaar 2012 | The definition of alcohol abuse/dependence is unclear. |
|  | Wasserman 1992 | A text book. |
|  | Wilcox 2004 | A review article rather than original article. |
|  | Wojnar 2009 | The severity of alcohol dependence on impulsive suicide attempt was investigated. |
|  | Yaldizli 2010 | The combined effect of alcohol use and other risk factors on suicide. |
|  | Zhang 2012 | Comparison of suicide death from other injuries in alcohol dependent individuals |
|  | Zhang 2012 | No association between alcohol and suicide was reported. |
|  | Zupanc 2013 | No comparison group. |
